# The impact of HLA-matching on reduced intensity conditioning regimen unrelated donor allogeneic stem cell transplantation for acute myeloid leukemia in patients above 50 years—a report from the EBMT acute leukemia working party

**DOI:** 10.1186/s13045-016-0295-9

**Published:** 2016-08-03

**Authors:** Marie T. Rubio, Bipin N. Savani, Myriam Labopin, Emmanuelle Polge, Dietger Niederwieser, Arnold Ganser, Rainer Schwerdtfeger, Gerhard Ehninger, Jürgen Finke, Arnold Renate, Charles Craddock, Nicolaus Kröger, Michael Hallek, Pavel Jindra, Mohamad Mohty, Arnon Nagler

**Affiliations:** 1 Department of Hematology, Hôpital Brabois, CHRU Nancy, Vandœuvre-lès-Nancy, France; 2 CNRS UMR 7365, IMoPA, Nancy, France; 3 Université de Lorraine, Nancy, France; 4 Acute Leukemia Working Party of EBMT, Paris, France; 5 Vanderbilt University Medical Center, Nashville, TN USA; 6 EBMT Paris study office/CEREST-TC, Paris, France; 7 Department of Haematology, Saint Antoine Hospital, Paris, France; 8 INSERM UMR 938, Paris, France; 9 Université Pierre et Marie Curie, Paris, France; 10 Division Hematology, Oncology and Hemostasiology, University Hospital Leipzig, Leipzig, Germany; 11 Department of Haematology, Hemostasis, Oncology and Stem Cell Transplantation, Hannover Medical School, Hannover, Germany; 12 Deutsche KlinikfürDiagnostik, KMT Zentrum, Wiesbaden, Germany; 13 Medizinische Klinik und Poliklinik I, Universitätsklinikum Carl Gustav Carus der TU Dresden, Dresden, Germany; 14 Department of Medicine, Hematology, Oncology, University of Freiburg, Freiburg, Germany; 15 MedizinischeKlinik m. S. Hämatologie/Onkologie, Campus Virchow Klinikum, Charité Universitätsmedizin Berlin, Berlin, Germany; 16 Centre for Clinical Haematology, Queen Elizabeth Hospital, Birmingham, UK; 17 Department of Stem cell Transplantation, University Hospital Eppendorf, Hamburg, Germany; 18 Department of Medicine, University of Cologne, Cologne, Germany; 19 Department of Hematology/Oncology, Charles University Hospital, Pilsen, Czech Republic; 20 Hematology Division, Chaim Sheba Medical Center, Tel Hashomer, Ramat Gan, Israel

**Keywords:** Allogeneic stem cell transplantation, Unrelated donor, Older patients, HLA matching, Acute leukemia, Toxicity, Anti-leukemic effect

## Abstract

**Background:**

Data comparing fully matched and mismatched-unrelated-donor (M- and mM-URD) allogeneic hematopoietic stem cell transplant (allo-SCT) following reduced intensity conditioning regimens for acute myeloid leukemia are limited.

**Methods:**

We retrospectively compared the outcome of 3398 patients above the age of 50 years who underwent 10/10 M-URD (*n* = 2567), 9/10 (*n* = 723), or 8/10 (*n* = 108) mM-URD allo-SCT for acute myeloid leukemia after reduced intensity conditioning regimen between 2000 and 2013. The Kaplan-Meier estimator, the cumulative incidence function, and Cox proportional hazards regression models were used where appropriate.

**Results:**

HLA matching had no impact on engraftment (*p* = 0.31). In univariate analysis, in comparison to 10/10 M-URD, mM-URD was associated with higher incidence of grade II–IV acute graft-versus-host disease (GVHD) (*p* = 0.0002), similar rates of chronic GVHD (*p* = 0.138) but increased incidence of its extensive form (*p* = 0.047). Compared to 10/10 M-URD, patients transplanted in the first complete remission (CR1) with a 9 or an 8/10 mM-URD had decreased 2-year leukemia free (LFS) (*p* = 0.005) and overall survivals (OS) (56.7, 46.1, and 50.2 %, respectively, *p* = 0.005), while outcomes were comparable between all groups for patients transplanted beyond CR1. In multivariate analysis, 9/10 versus 10/10 URD was associated with higher non-relapse mortality (HR 1.34, *p* = 0.001), similar risk of relapse and chronic GVHD and inferior LFS (HR 1.25, *p* = 0.0001), and OS (HR 1.27, *p* = 0.0001). There was no difference in adjusted transplant outcomes between 9/10 and 8/10 mM-URD.

**Conclusions:**

Reduced intensity conditioned allo-SCT with a 10/10 M-URD remains the preferable option for AML patients above the age of 50 years. The use of a 9/10 or an 8/10 mM-URD in patients not having a fully matched donor represents an alternative therapeutic option that should be compared to other alternative donor transplant strategies.

**Electronic supplementary material:**

The online version of this article (doi:10.1186/s13045-016-0295-9) contains supplementary material, which is available to authorized users.

## Background

The development of reduced intensity conditioning (RIC) regimens has allowed to offer allogeneic hematopoietic stem cell transplantation (allo-SCT) to adults above the age of 50 years and patients with comorbidities [1–8]. In acute myeloid leukemia (AML), allo-SCT performed with RIC regimen improves the leukemia-free survival (LFS) of older adults in comparison to standard chemotherapy [9–11] and reduces non-relapse mortality (NRM) in comparison to myeloablative conditioning (MAC) [11, 12]. RIC allo-SCT is therefore the treatment of choice for intermediate- and high-risk AML patients above 50 years having an HLA compatible donor [9–11, 13].

As only about one third of the patients that are in need of allo-SCT have a matched-related donor and donor registries have increased the probability to find a suitable unrelated donor (URD), increasing numbers of patients are receiving matched (M-URD) and mismatched unrelated donor (mM-URD) allo-SCT [14, 15]. The outcomes of M-URD and mM-URD transplant have significantly improved in the last decade [14, 16, 17]. Although the MRC AML15 Trial has recently reported improved overall survival with matched related donors (MRD) compared to M-URD due to reduced NRM [11], several studies have shown similar outcomes of MRD in comparison to M-URD or mM-URD after RIC allo-SCT for AML [6, 18–20]. Among M-URD, comparative data between M-URD and mM-URD are limited, in particular in the context of RIC allo-SCT for older adults with AML.

Acute leukemias in the elderly population are characterized as more aggressive diseases due to selection of more primitive clones with higher prevalence of complex karyotype and presence of multidrug resistance [21, 22]. Disease control after RIC allo-SCT mainly relies on the anti-leukemic effect of allogeneic NK and T cells [23, 24]. Since HLA mismatching might improve graft-versus-leukemia effect in high-risk acute leukemias [25], one might hypothesize that HLA-mismatched RIC-allo SCT for AML in patients above the age of 50 years could offset the higher risk of NRM by reducing the need of myeloablative doses of chemotherapy aiming in reducing the risk of relapse. In the present study, we analyzed the outcome of 3398 adult patients above the age of 50 years who underwent matched or mismatched URD allo-SCT with RIC regimen for AML.

## Methods

### Study design and data collection

This is a retrospective multicenter analysis using the data set of the acute leukemia working party (ALWP) of the European Society of Blood and Marrow Transplantation (EBMT) group registry. The EBMT is a voluntary working group of more than 500 transplant centers that are required to report all consecutive stem cell transplantations and follow-ups once a year. Audits are routinely performed to determine the accuracy of the data. The study was planned and approved by the ALWP of the EBMT. In addition, the study protocol was approved by the institutional review board at each site and complied with country-specific regulatory requirements. The study was conducted in accordance with the Declaration of Helsinki and Good Clinical Practice guidelines. Since 1990, patients provide informed consent authorizing the use of their personal information for research purposes. Eligibility criteria for this analysis included patients with AML above 50 years old who underwent a first allo-SCT from an HLA-matched (10/10) or mismatched (9/10 or 8/10) unrelated donor (mM-URD) following RIC regimen between 2000 and 2013. All donors were HLA matched (10/10) or mismatched at one or two loci (9/10 or 8/10) (−A, −B, −C, DRB1, −DQB1). HLA typing was determined at all loci by high-resolution techniques. Exclusion criteria were previous allogeneic or cord blood transplantation, ex vivo T cell-depleted stem cell graft. Regimens were classified as RIC based on EBMT criteria [26]. Variables collected included recipient and donor characteristics (age, gender, CMV serostatus, recipient’s Karnofsky status at transplant), disease characteristics and status at transplant, year of transplantation and interval from diagnosis to transplantation, transplant-related factors including conditioning regimen, pre-transplant in vivo T cell depletion, stem cell source (bone marrow (BM) or peripheral blood (PB)), post-transplant graft-versus-host disease (GVHD) prophylaxis, and outcome variables (acute and chronic GVHD, relapse, NRM, LFS, OS, and causes of death). Grading of acute GVHD was performed using established criteria [27]. Chronic GVHD was classified as limited or extensive according to published criteria [28]. For the purpose of this study, all necessary data were collected according to the EBMT guidelines, using the EBMT Minimum Essential Data forms. The list of institutions reporting data included in this study is provided in Additional file 1: Table S1.

### Statistical analysis

Primary endpoints of the study were incidences and severity of acute and chronic GVHD, NRM, and disease relapse incidence (RI). Secondary endpoints included engraftment, OS, and LFS. Start time was the date of transplant for all endpoints. LFS was defined as survival without relapse or progression and NRM as death without relapse/progression. Cumulative incidence functions (CIFs) were used to estimate RI and NRM in a competing risk setting, because death and relapse compete with each other. For estimating the cumulative incidence of chronic GVHD, we considered relapse and death to be competing events. The three groups according to level of HLA matching were compared by the chi-square method for qualitative variables, whereas the Mann–Whitney test was applied for continuous parameters. Univariate comparisons were done using the log-rank test for OS, LFS, and the Gray’s test for RI, NRM, and GVHD cumulative incidences. Multivariate analyses were performed using logistic regression for grade II–IV acute GVHD rate and Cox proportional hazards model for all other endpoints. Factors differing in terms of distribution between the three groups and all factors known as potentially risk factors were included in the final model. Factors included in the Cox models included HLA matching, patient age (analyzed by 10-year scale), and Karnofsky status (≥80 % versus below 80 %), year of transplantation, time from diagnosis to transplantation (per 6 months), disease status at transplantation, secondary AML versus de novo AML, low-dose TBI-based versus chemotherapy-based RIC regimens, use of in vivo T cell depletion, female donor to male recipient versus other gender combinations, and CMV risk (high-risk seropositive recipient with seronegative donor versus others combinations). All tests were two sided. The type I error rate was fixed at 0.05 for determination of factors associated with time to event outcomes. Statistical analyses were performed with SPSS 22.0 (IBM Corp., Armonk, NY, USA) and R 3.1.1 software packages (R Development Core Team, Vienna, Austria).

## Results

### Patient, disease, and transplant characteristics

Details of patients, disease, and transplant characteristics are summarized in Table 1. Three thousand three hundred ninety-eight patients with AML were included in the study. Two thousand five hundred sixty-seven patients (75.5 %) received a HLA 10/10 fully matched, while 723 (21.3 %) received a 9/10 and 108 (3.2 %) received an 8/10 mismatched unrelated donor (mM-URD) allo-SCT between 2000 and 2013. All patients were older than 50 years and median age was comparable between the three groups (60 to 61 years with ranges between 50 and 78 years) (Table 1). Patients receiving an HLA 8/10 mM-URD had been transplanted more lately than the two other groups (median year of transplantation 2009 versus 2011, *p* = 0.001) and had a longer follow up of surviving patients (median 34 versus 24 months, *p* = 0.042). Interval from diagnosis to transplantation was shorter in the HLA 10/10 in comparison to the 9/10 and 8/10 mM-URD groups (212 versus 250 and 295 days, respectively, *p* = 0.0001). Patients in the mM-URD groups had been more frequently transplanted with a female donor (*p* < 10^−4^) and more transplants were performed from female donor to male recipient (*p* = 0.01). Secondary AML was more frequent in the 8/10 mM-URD group (44 versus <30 %, *p* = 0.001). The proportions of poor cytogenetics were equally distributed between the three groups although cytogenetic analysis was missing in 52 to 55 %, of the patients. Significantly higher numbers of patients were transplanted in CR1 in the 10/10 URD in comparison to the 9/10 and 8/10 mM-URD groups (55 versus 46.1 and 44.4 %, respectively, *p* = 0.0002). Peripheral blood represented the major source of stem cells in all groups. TBI-based RIC was less frequently used in the 8/10 HLA mM-URD group (*p* = 0.02). Patients in the mM-URD groups had received more frequently in vivo T cell depletion in comparison to 10/10 matched URD SCT (86 % in 8/10, 83.1 % in 9/10 versus 73.5 % in 10/10 groups, *p* < 10^−4^). There was higher proportions of patients with high CMV reactivation risk (negative donor with positive recipient CMV serologies) in the 9/10 group in comparison to the others (*p* = 0.029). The choice of conditioning and GVHD prophylaxis was dependent on centers’ protocols and strategies of transplantation.

**Table 1:** Patients and disease characteristics

Patient characteristics	HLA 10/10	HLA 9/10	HLA 8/10	*p* value
Number of patients	2567	723	108	
Recipient age at SCT (years, range)	61 (50–78)	61 (50–77)	60 (50–73)	0.584
Recipient gender, *n* (%)				0.707
Male	1386 (54 %)	387 (53.7 %)	62 (58 %)	
Female	1177 (46 %)	334 (46.3 %)	45 (42 %)	
Year of SCT (median), year (%)	2011 (00–13)	2011 (02–13)	2009 (00–13)	0.001
Interval from diagnosis to SCT (days)	212	250	295	0.0001
Median follow-up^a^ (months, range)	24 (1–150)	24 (1–139)	34 (3–117)	0.042
Donor age (years, range)	33 (16–61)	36 (20–61)	35 (20–55)	0.02
Donor gender, *n* (%)				<10^−4^
Male	1845 (73 %)	463 (65.2 %)	69 (64.5 %)	
Female	682 (27 %)	247 (34.8 %)	38 (35.5 %)	
Female donor to male recipient, *n* (%)	281 (11.1 %)	106 (15 % )	17 (16 % )	0.01
Diagnosis, *n* (%)				0.001
De novo AML	1805 (70.3 %)	527 (72.9 %)	60 (55.6 %)	
Secondary AML	762 (29.7 %)	196 (27.1 %)	48 (44.4 %)	
Cytogenetics in de novo AML, *n* (% of available data)				0.235
Good	75 (9.2 %)	15 (5.9 %)	2 (7.1 %)	
Intermediate	545 (67.2 %)	183 (72 %)	23 (82 %)	
Poor	191 (23.5 %)	56 (22 %)	3 (10.7 %)	
Not available/failed	994 (55 %)	273 (51.8 %)	32 (53.3 %)	
Disease status at SCT, *n* (%)				0.0002
CR1	1413 (55 %)	333 (46.1 %)	48 (44.4 %)	
≥CR2	504 (19.7 %)	171 (23.6 %)	25 (23.2 %)	
Active disease	650 (25.3 %)	219 (30.3 %)	35 (32.4 %)	
Source of SC, *n* (%)				0.173
BM	154 (6 %)	56 (7.8 %)	9 (8.3 %)	
PB	2413 (94 %)	667 (92.2 %)	99 (91.7 %)	
Conditioning, n (%)				0.02
Chemo alone	1836 (71.5 %)	550 (76.1 %)	84 (77.8 %)	
Low TBI	731 (28.5 %)	173 (23.9 %)	24 (22.2 %)	
In vivo T cell depletion, *n* (%)				<10^−4^
No	672 (26.4 %)	121 (16.9 %)	15 (14.0 %)	
ATG	1460 (57.5 %)	468 (65.2 %)	59 (55.1 %)	
Campath	409 (16.1 %)	129 (18.0 %)	33 (30.8 %)	
Post-transplant GVHD prophylaxis				0.07
CsA	504 (19.9 %)	135 (18.8 %)	27 (25.5 %)	
CsA/FK 506 + MTX	517 (20.4 %)	177 (24.7 %)	16 (15.1 %)	
CsA/FK 506 + MMF	1353 (53.4 %)	353 (49.2 %)	53 (50 %)	
Other	160 (6.3 %)	53 (7.4 %)	10 (9.4 %)	
Missing	33	5	2	
Karnosky at SCT, *n* (%)				0.47
≤80 %	173 (6.7 %)	42 (5.8 %)	9 (8.4 %)	
>80 %	2231 (86.9 %)	641 (88.7 %)	91 (84.2 %)	
Missing	163 (6.4 %)	40 (5.5 %)	8 (7.4 %)	
Patient positive CMV serology, *n* (%)	1634 (64.9 %)	492 (69.1 %)	67 (63.8 %)	0.10
CMV risk, *n* (%)				0.029
Low	695 (27.8 %)	155 (21.9 %)	26 (24.8 %)	
Intermediate	1072 (42.8 %)	316 (44.7 %)	47 (44.8 %)	
High	734 (29.4 % )	236 (33.4 %)	32 (30.5 %)	

*AML* acute myeloid leukemia, *ATG* anti-thymocyte globulin, *BM* bone marrow, *CMV* cytomegalovirus, *CMV risk* low = negative recipient and donor serology, *high* positive recipient and negative donor serology, *intermediate* all other combinations, *CR* complete remission, *PB* peripheral blood, *SC* stem cells, *SCT* stem cell transplantation

^a^For patients alive

### Engraftment and GVHD

Engraftment and incidences of acute and chronic GVHD are summarized in Table 2. There was no difference in terms of engraftment between the 10/10, 9/10, and 8/10 groups (97.3, 96.3, and 97.1 %, respectively, *p* = 0.313). Median time for ANC > 0.5 × 10^9^/L was also similar between the three groups (16, 16, and 15.5 days, respectively, *p* = 0.538).

**Table 2:** Engraftment and GVHD

	HLA 10/10	HLA 9/10	HLA 8/10	*p* value
Total number of patients	2567	723	108	
Engraftment, *n* (%)	2458 (97.3 %)	678 (96.3 %)	101 (97.12 %)	0.313
No engraftment, *n* (%)	67 (2.7 %)	26 (3.7 %)	3 (2.9 %)	
Missing, *n*	42	19	5	
Median time ANC >0.5 G/L (days, range)	16 (0–103)	16 (1–165)	15.5 (1–33)	0.538
Acute GVHD,				
Grade 0–I, *n* (%)	1826 (74.4 %)	469 (67.2 %)	67 (65.7 %)	0.0002
Grades II–IV, *n* (%)	629 (25.6 %)	229 (32.8 %)	35 (34.3 %)	
Grades III–IV, *n* (%)	234 (9.5 %)	90 (12.9 %)	11 (10.8 %)	0.035
Missing, *n*	75	21	3	
Chronic GVHD^a^				
All grades	35.0 % [32.9–37.2]	35.1 % [31.2–39]	44.4 % [33.6–54.6]	0.138
Extensive	17.1 % [15.4–18.9]	15.2 % [12.3–18.5]	26.1 % [16.8–36.4]	0.047
Limited, *n*	369	109	14	
Extensive, *n*	324	80	21	
Missing, *n*	38	18	5	

*GVHD* graft-versus-host disease

^a^Two-year cumulative incidence

**Fig. 1:**
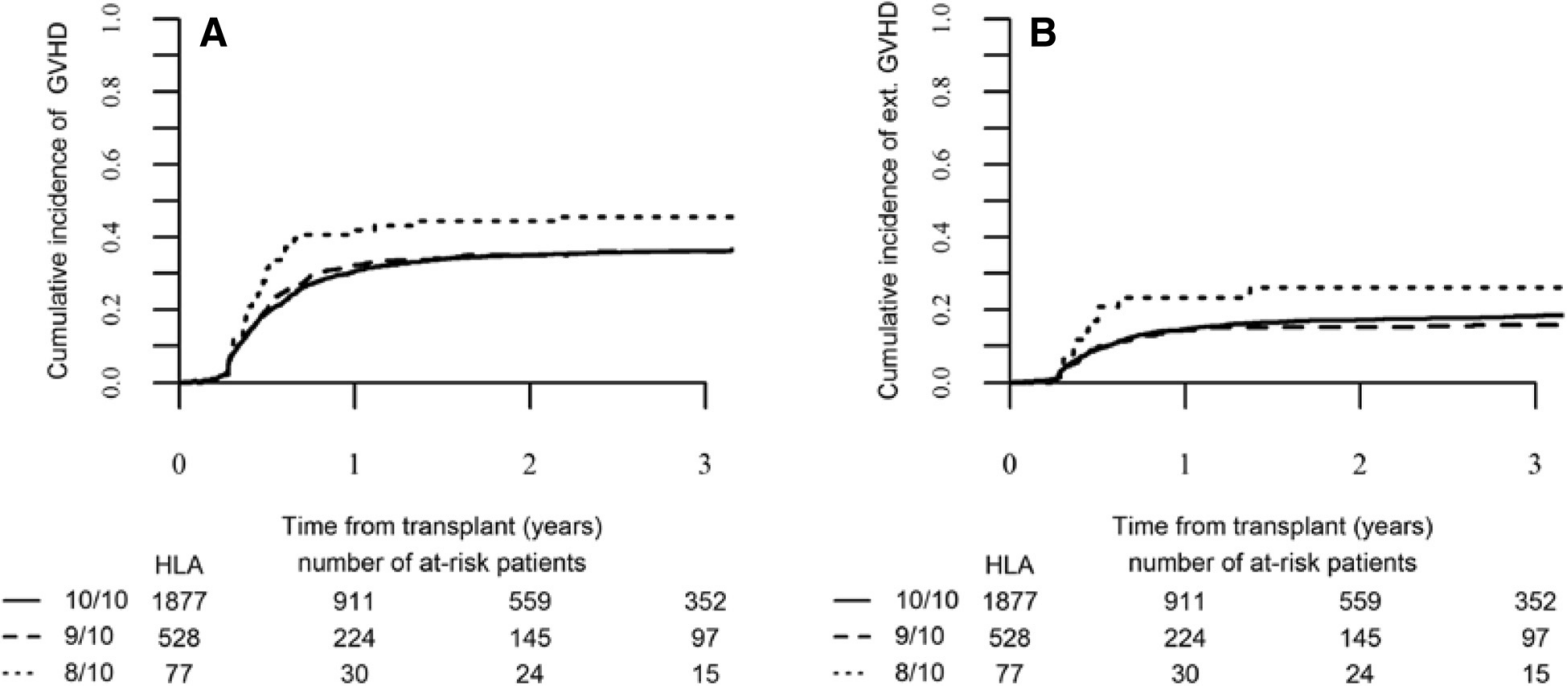
Chronic GVHD according to HLA-matching. **a** Cumulative incidence of global chronic (c) GVHD and **b** of extensive chronic GVHD in the HLA-matched and mismatched-URD groups as mentioned (global *p* value = 0.138 for all cGVHD and *p* = 0.047 for extensive cGVHD)

In univariate analysis, incidence of day 100 grade II–IV and grade III–IV acute GVHD were significantly higher in 8/10 and 9/10 mM-URD in comparison to HLA 10/10 M-URD groups (34.3, 32.8, and 25.6 % for grade II–IV aGVHD, respectively, *p* = 0.0002; and 10.8, 12.9, and 9.5 % for grade III–IV aGVHD, respectively, *p* = 0.035) (Table 2). Incidences of grade II–IV and grade III–IV aGVHD were lower in patients who received in vivo T cell depletion compared to those who did not (23.7 versus 33 %, *p* < 10^−4^, and 9 versus 14.3 %, p < 10^−4^, respectively). Two-year incidence of all grades chronic GVHD was not significantly different between the three groups: 44.5 % in the HLA 8/10 mM-URD, 35.1 % in the 9/10 mM-URD, and 35 % in the HLA 10/10 M-URD groups (*p* = 0.138) (Tables 2 and 3 and Fig. 1a). However, an increased incidence was observed in the HLA 8/10 mM-URD group for patients transplanted above second complete remission (≥CR2) (65.5 versus 34.7 % in HLA 9/10 and 35 % in HLA 10/10 matched mM-URD groups, *p* = 0.01) (Table 3). Incidence of extensive chronic GVHD was also increased in the 8/10 mM-URD compared to 9/10 mM-URD and 10/10 M-URD groups (26.1 versus 15.2 and 17.1 %, respectively, *p* = 0.047) (Table 2 and Fig. 1b), in particular in the group of patients transplanted in advanced phase (32.2 versus 18.3 and 13.2 %, respectively, *p* = 0.02) (Table 3). Incidences of overall cGVHD and extensive cGVHD were reduced in patients who received an in vivo T cell depletion in comparison to those who did not (32.9 versus 45 %, *p* < 0.0001, and 14.6 versus 26.4 %, *p* < 0.0001, respectively) (Table 3). As shown in Table 4, GVHD-related deaths represented 17.2, 20.1, and 17.3 % of all causes of death in the 8/10, 9/10, and 10/10 HLA groups, respectively.

**Table 3:** Comparison of 2-year outcomes according to donor HLA matching, disease status, and use of in vivo T cell depletion

Disease status	Patients group and *p* value	RI	NRM	LFS	OS	cGVHD	Extensive cGVHD
All	10/10	30.1 % [28.2–32.1]	24.2 % [16.4–32.9]	45.6 % [43.5–47.7]	50.6 % [48.5–52.8]	35.0 % [32.9–37.2]	17.1 % [15.4–18.9]
	9/10	32.5 % [28.9–36.3]	31.6 % [23–40.5]	35.8 % [32–39.7]	41.3 % [37.3–45.3]	35.1 % [31.2–39]	15.2 % [12.3–18.5]
	8/10	24.2 % [16.4–32.9]	35.5 % [26.6–44.4]	40.3 % [30.6–50]	43.5 % [33.6–53.3]	44.4 % [33.6–54.6]	26.1 % [16.8–36.4]
	*p* value	0.152	0.002	0.0001	0.0001	0.138	0.047
CR1	10/10	24.8 % [22.4–27.3]	22.5 % [11.6–35.7]	52.6 % [49.7–55.5]	56.7 % [53.8–59.6]	37.6 % [34.7–40.5]	18.1 % [15.8–20.5]
	9/10	31.6 % [26.3–37.2]	26.8 % [14.8–40.2]	41.6 % [35.7–47.5]	46.1 % [40.1–52.2]	35.9 % [30–41.9]	13 % [9.1–17.7]
	8/10	17.7 % [8.1–30.2]	33.3 % [20.4–46.8]	49% [34.3–63.7]	50.2 % % [35.2–65.1]	43.4 % [26.4–59.2]	22.1 % [9.5–37.9]
	*p* value	0.010	0.136	0.005	0.005	0.641	0.107
≥CR2	10/10	32.6 % [28.2–37.1]	24.1 % [8.7–43.5]	43.3 % [38.5–48.1]	50.1 % [45.3–55]	35 % [30.3–39.7]	13.9 % [10.6–17.7]
	9/10	26.3 % [19.5–33.5]	32.5 % [14.6–51.8]	41.2 % [33.3–49.2]	48 % [40–56.1]	34.7 % [26.8–42.7]	18.3 % [12.1–25.6]
	8/10	22.4 % [7.7–41.7]	24.6 % [9.1–44.1]	53 % [31.3–74.7]	62 % [40.8–83.3]	65.5 % [39.6–82.4]	32.2 % [13.8–52.3]
	*p* value	0.304	0.089	0.290	0.253	0.010	0.020
Act. dis.	10/10	39.7 % [35.7–43.7]	28 % [13.8–44]	32.3 % [28.4–36.3]	37.9 % [33.8–42]	29.4 % [25.5–33.4]	17.5 % [14–21.5]
	9/10	38.4 % [31.6–45.3]	38 % [22.3–53.6]	23.6 % [17.4–29.7]	29.1 % [22.5–35.7]	33.9 % [27.1–40.8]	16.1 % [10.7–22.5]
	8/10	34.3 % [19–50.2]	45.7 % [29.7–60.4]	20 % [6.7–33.3]	22.9 % [8.9–36.8]	30 % [14.4–47.4]	26.1 % [10.1–45.5]
	*p* value	0.902	0.062	0.107	0.139	0.587	0.578
In vivo	No	28.8 % [25.4–32.3]	27.1 % [23.7–30.7]	44 % [40.1–48]	47.7 % [43.7–51.8]	45 % [40.8–49]	26.4 % [22.5–30.4]
T cell	Yes	30.7 % [28.8–32.6]	25.9 % [22.5–29.4]	43.4 % [41.3–45.5]	48.8 % [46.7–50.9]	32.9 % [30.9–34.9]	14.6 % [13–16.2]
Depletion	*p* value	0.920	0.448	0.613	0.448	1.0483e-05	1.337e-09

*Act. dis.* active disease, *cGVHD* chronic graft-versus-host-disease, *CR* complete remission, *LFS* leukemia-free survival, *NRM* non relapse mortality, *OS* overall survival, *RI* relapse incidence

**Table 4:** Causes of death according to donor HLA matching

	HLA 10/10	HLA 9/10	HLA 8/10
Relapse	552 (47.5 %)	173 (43.4 %)	21 (36.2 %)
Infection	249 (21.3 %)	100 (25.1 %)	21 (36.2 %)
GVHD	202 (17.3 %)	80 (20.1 %)	10 (17.2 %)
Graft failure/rejection	11 (0.9 %)	1 (0.3 %)	0
Cardiac toxicity	8 (0.7 %)	4 (1 %)	1 (1.7 %)
Haemorhage	14 (1.2 %)	5 (1.3 %)	1 (1.7 %)
VOD	12 (1 %)	8 (2 %)	0
Idiopathic pneumonia	25 (2.1 %)	9 (2.3 %)	0
Second malignancy	24 (2.1 %)	5 (1.3 %)	0
Other SCT-related	70 (6 %)	14 (3.5 %)	4 (6.9 %)

*GVHD* graft-versus-host disease, *SCT* stem cell transplantation, *VOD* veno-occlusive disease

Multivariate analyses are shown in Table 5. Factors associated with increased risk of grade II–IV acute GVHD were the use of 9/10 mM-URD in comparison to 10/10 M-URD (HR 1.47, 95 % CI 1.21–1.79; *p* = 0.0001), active disease at transplantation (HR 1.34, 95 % CI 1.1–1.63; *p* = 0.004) and high CMV risk (seropositive recipient and seronegative donor) (HR 1.28, 95 % CI 1.03–1.60; *p* = 0.028), while the use of in vivo T cell depletion was associated with reduced risk of grade II–IV acute GVHD (HR 0.61, 95 % CI 0.49–0.75; *p* < 10^−4^). The use of an 8/10 mM-URD was not associated with an increased incidence of grade II–IV aGVHD in comparison to a 9/10 mM-URD (*p* = 0.952). Chronic GVHD was not significantly different between HLA 10/10 matched and 9/10 mM-URD groups (*p* = 0.418) or between HLA 9/10 and 8/10 mM-URD groups (*p* = 0.145). The only factor associated with increased risk of chronic GVHD was active disease at allo-SCT (HR 1.22, 95 % CI 1.03–1.45; *p* = 0.019), while in vivo T cell depletion was associated with reduced risk of chronic GVHD development (HR 0.72, 95 % CI 0.61–0.85; *p* = 0.0001) (Table 5).

**Table 5:** Multivariate analysis

	Relapse		NRM		Acute GVHD		Chronic GVHD		LFS		OS	
	*p* value	HR (95 % CI)	*p* value	HR (95 % CI)	*p* value	OR (95 % CI)	*p* value	HR (95 % CI)	*p* value	HR (95 % CI)	*p* value	HR (95 % CI)
HLA 10/10 (ref)		1.00		1.00		1.00		1.00		1.00		1.00
HLA 9/10 versus 10/10	0.038	1.18 (1.01–1.38)	0.001	1.34 (1.13–1.58)	0.0001	1.47 (1.21–1.79)	0.418	1.07 (0.91–1.26)	0.0001	1.25 (1.11–1.40)	0.0001	1.27 (1.13–1.44)
HLA 8/10 versus 9/10	0.064	0.67 (0.43–1.02)	0.398	1.17 (0.81–1.68)	0.952	1.01 (0.63–1.62)	0.145	1.31 (0.91–1.88)	0.432	0.90 (0.68–1.18)	0.557	0.92 (0.69–1.22)
Age at SCT (10 years)	0.701	1.02 (0.91–1.15)	<10^−5^	1.41 (1.24–1.59)	0.465	1.05 (0.91–1.22)	0.501	1.04 (0.93–1.17)	0.0001	1.18 (1.09–1.29)	<10^−5^	1.25 (1.14–1.37)
Interval diag. to SCT^a^	0.007	0.96 (0.93–0.99)	0.636	1.01 (0.98–1.03)	0.306	0.98 (0.95–1.02)	0.833	1.00 (0.97–1.02)	0.108	0.98 (0.96–1.00)	0.122	0.98 (0.96–1.00)
Disease status at SCT												
CR1 (ref)		1.00		1.00		1.00		1.00		1.00		1.00
CR2 versus CR1	4.10^−5^	1.50 (1.24–1.82)	0.713	1.04 (0.85–1.28)	0.728	1.04 (0.82–1.32)	0.558	1.06 (0.88–1.28)	0.002	1.25 (1.09–1.44)	0.016	1.20 (1.03–1.39)
Act.dis. versus CR1	<10^−5^	2.06 (1.76–2.42)	0.001	1.35 (1.14–1.60)	0.004	1.34 (1.1–1.63)	0.019	1.22 (1.03–1.45)	<10^−5^	1.69 (1.51–1.90)	<10^−5^	1.63 (1.44–1.84)
Secondary AML	0.699	0.97 (0.83–1.13)	0.0004	1.32 (1.13–1.54)	0.418	1.08 (0.90–1.30)	0.196	1.11 (0.95–1.29)	0.029	1.13 (1.01–1.26)	0.021	1.14 (1.02–1.28)
Karnofsky ≥80 %	0.149	0.83 (0.64–1.07)	<10^−5^	0.55 (0.43–0.70)	0.838	0.97 (0.70–1.33)	0.318	0.86 (0.64–1.16)	10^−5^	0.68 (0.57–0.81)	<10^−5^	0.63 (0.58–0.76)
TBI	0.005	1.26 (1.07–1.48)	0.767	1.03 (0.85–1.24)	0.088	0.83 (0.67–1.03)	0.610	1.04 (0.88–1.24)	0.019	1.16 (1.02–1.30)	0.022	1.16 (1.02–1.32)
In vivo T cell depletion	0.483	1.07 (0.89–1.27)	0.253	0.90 (0.74–1.08)	<10^−5^	0.61 (0.49–0.75)	0.0001	0.72 (0.61–0.85)	0.790	0.98 (0.86–1.12)	0.72	0.98 (0.85–1.12)
Female D to male R	0.112	0.84 (0.68–1.04)	0.172	1.15 (0.94–1.42)	0.063	1.26 (0.99–1.61)	0.078	1.19 (0.98–1.45)	0.846	0.99 (0.85–1.14)	0.896	1.01 (0.87–1.18)
High CMV risk (R+/D−)	0.665	1.04 (0.87–1.24)	0.002	1.35 (1.11–1.65)	0.028	1.28 (1.03–1.60)	0.814	0.98 (0.82–1.17)	0.019	1.17 (1.03–1.33)	0.001	1.25 (1.09–1.43)

*Act.dis.* active disease, *CR* complete remission, *D* donor, *D−* donor, *CMV* serology, *GVHD* graft-versus-host-disease, *LFS* leukemia-free survival, *NRM* non relapse mortality, *OS* overall survival, *PB* Peripheral blood, *Ref* reference, *R* recipient, *R+* positive recipient, *CMV* serology, *SCT* allogeneic stem cell transplantation, *TBI* total body irradiation

^a^Analyzed per 6-month interval

### Toxicity and NRM

Two-year NRM for the entire cohort was 26.1 % (95 % CI, 24.6–27.8). In univariate analysis, 2-year NRM was significantly higher in mM-URD groups (35.5 %, 95 % CI 26.6–44.4 in HLA 8/10 and 31.6 %, 95 % CI 23–40.5 in HLA 9/10 mM-URD groups) in comparison to the HLA 10/10 M-URD group (24.2 %, 95 % CI 16.4–32.9) (*p* = 0.001) (Table 3). In multivariate analysis, the use of a 9/10 mM-URD was associated with increased NRM in comparison to HLA 10/10 matched URD (HR 1.34, 95 % CI 1.13–1.58; *p* = 0.001), while there was no difference in NRM between 9/10 and 8/10 mM-URD (*p* = 0.398) (Table 5). The other factors associated with higher NRM were age at allo-SCT (HR 1.41, 95 % CI 1.24–1.59; *p* < 10^−5^); active disease (HR 1.35, 95 % CI 1.14–1.60; *p* = 0.001), secondary AML (HR 1.32, 95 % CI 1.13–1.54; *p* = 0.0004) and high CMV risk (HR 1.35, 95 % CI 1.11–1.65; *p* = 0.002). Karnofsky performance status at allo-SCT above 80 % was associated with reduced NRM (HR 0.55, 95 % CI 0.43–0.70; *p* < 10^−5^) (Table 5).

The main causes of NRM were infectious complications and GVHD (Table 4). Death from infection was reported in 21, 100, and 249 patients and represented 51.2, 41.7, and 36.1 % of the causes of NRM in the HLA 8/10, 9/10, and 10/10 groups, respectively. Death from GVHD occurred in 10, 80, and 202 patients, representing 24.4, 33.3, and 29.3 % of the causes of NRM in the 8/10, 9/10, and 10/10 groups, respectively. Death from organ toxicity was low and represented <10 % of the causes of NRM in the three groups, in particular death related to sinusoidal obstructive syndrome (SOS) concerned 0, 8, and 12 patients (0, 0.4, and 1.7 % of causes of NRM) in the HLA 8/10, 9/10, and 10/10 groups, respectively.

### Relapse

**Fig. 2:**
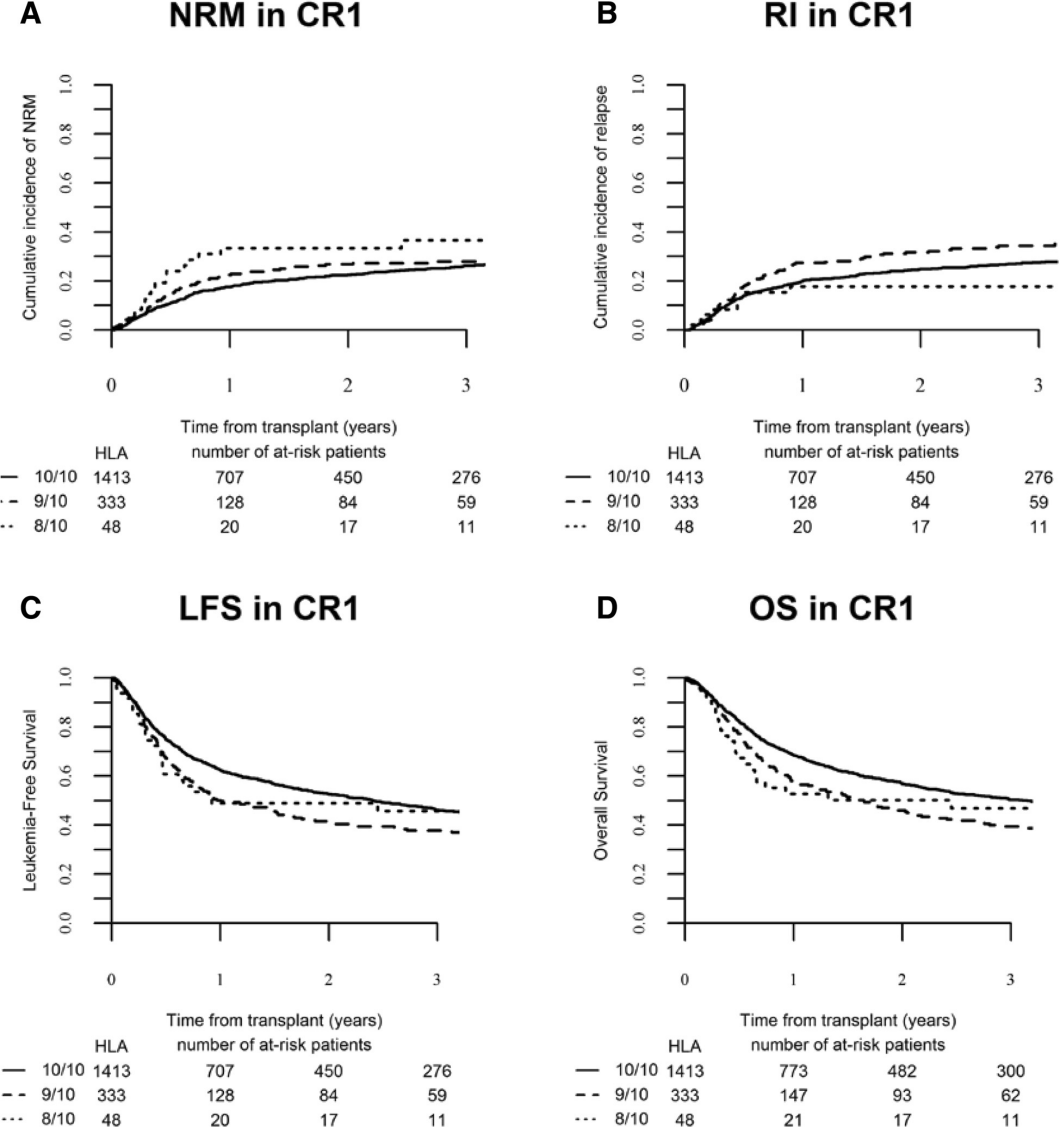
Transplant outcomes according to HLA-matching in patients transplanted in CR1. **a** Cumulative incidence of non-relapse mortality (NRM) (global *p* value = 0.136), **b** cumulative incidence of relapse (global *p* value = 0.01), **c** leukemia-free survival (global *p* value = 0.005), and **d** overall survival (global *p* value = 0.005) in the different HLA-matched and mismatched-URD groups as mentioned

**Fig 3:**
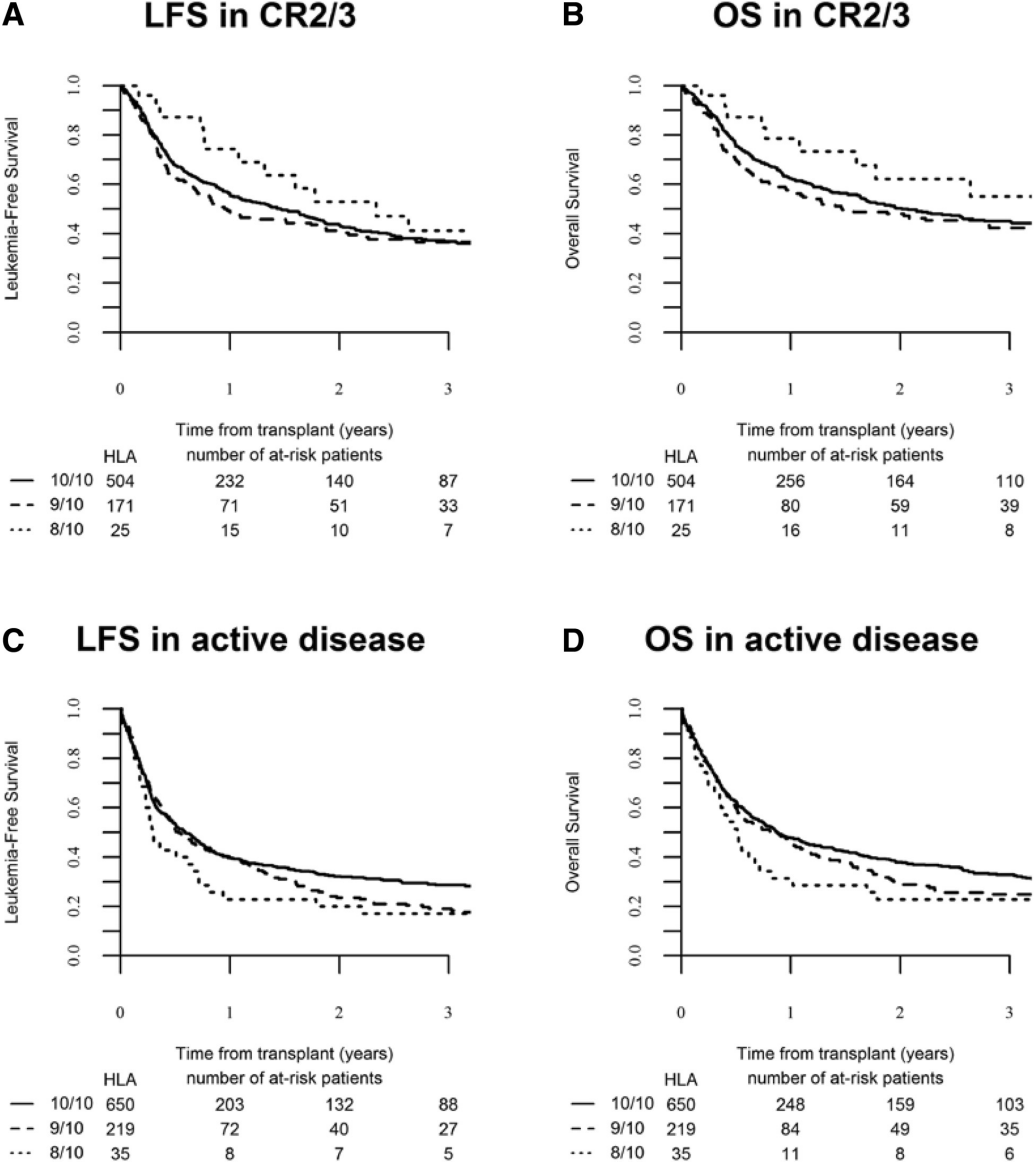
Transplant outcomes according to HLA-matching in patients transplanted with advanced diseases. **a** Leukemia-free survival (global *p* value = 0.290) and **b** overall survival (global *p* value = 0.253) of patients transplanted in CR2 and CR3 in the different HLA-matched and mismatched-URD groups as mentioned. **c** Leukemia-free survival (global *p* value = 0.107) and **d** overall survival (global *p* value = 0.139) of patients transplanted with active disease in the different HLA-matched and mismatched-URD groups as mentioned

Cumulative RI at 2 years was 30.4 % (95 % CI 28.8–32.1). In univariate analysis, RI was 24.2 % (95 % CI 16.4–32.9), 32.5 % (95 % CI 28.9–36.3), and 30.1 % (95 % CI 28.2–32.1) in the HLA 8/10, 9/10, and 10/10 URD groups, respectively (*p* = 0.152) (Table 3). Recurrence of original disease represented the first cause of death in the three groups of patients (Table 4). When analyzed according to disease status, in univariate analysis, RI was different between the three HLA typing groups for patients transplanted in CR1: 17.7 % (95 % CI 8.1–30.2) in the 8/10 mM-URD versus 31.6 % (95 % CI 26.3–36.2) in the 9/10 and 24.8 % (95 % CI 22.4–27.3) in the 10/10 URD group (*p* = 0.01) (Table 3 and Fig. 2b). Relapse incidence was similar between the three groups in more advanced diseases (Table 3 and Fig. 3b). In multivariate analysis, in comparison to 10/10 M-URD, the use of a 9/10 mM-URD was associated with an increased risk of relapse (HR 1.18, 95 % CI 1.01–1.38; *p* = 0.038) and there was a trend for reduced risk of relapse with the use of an 8/10 mM-URD (HR 0.67, 95 % CI 0.43–1.02; *p* = 0.064) (Table 5). Other factors associated with higher risk of relapse were advanced disease (≥CR2) (HR 1.50, 95 % CI 1.24–1.82; *p* = 4.10^−5^); active disease at allo-SCT (HR 2.06, 95 % CI 1.76–2.42; *p* < 10^−5^) and the use of low-dose TBI-based RIC (HR 1.26, 95 % CI 1.07–1.48; *p* = 0.005). Shorter interval from diagnosis to allo-SCT was associated with reduced RI (HR 0.96, 95 % CI 0.93–0.99; *p* = 0.007) (Table 5). Of note, the use of in vivo T cell depletion had no impact on RI in both univariate and multivariate analyses (Tables 3 and 5).

### Leukemia-free survival

Overall LFS at 2 years was 43.3 % (95 % CI 41.5–45.2). In univariate analysis, overall 2-year LFS was significantly higher in HLA 10/10 M-URD group (45.6 %, 95 % CI 43.5–47.7) in comparison to mM-URD groups (35.8 %, 95 % CI 32–39.7 in HLA 9/10 and 40.3 %, 95 % CI 30.6 50 in HLA 8/10 mM-URD groups) (*p* = 0.0001) (Table 3). Improved LFS with HLA 10/10 M-URD versus 9/10 and 8/10 mM-URD groups was observed in patients transplanted in CR1 (52.6 %, 95 % CI 49.7–55.5 versus 41.6 %, 95 % CI 35.7–47.5 and 49 %, 95 % CI 34.3–63.7, respectively, *p* = 0.005) but not in patients transplanted in advanced stage diseases (≥CR2 and active disease) (Table 3, Figs. 2c and 3c). In multivariate analysis, the use of a 9/10 mM-URD was associated with reduced LFS in comparison to HLA 10/10 M-URD (HR 1.25, 95 % CI 1.11–1.40; *p* = 0.0001), while there was no difference between 9/10 and 8/10 mM-URD (*p* = 0.432) (Table 5). The other factors associated with shorter LFS were age at SCT (HR 1.18, 95 % CI 1.09–1.29; *p* = 0.0001); disease status ≥ CR2 at SCT (HR 1.25, 95 % CI 1.09–1.44; *p* = 0.002); active disease at SCT (HR 1.69, 95 % CI 1.51–1.90; *p* < 10^−5^); secondary AML (HR 1.13, 95 % CI 1.01–1.26; *p* = 0.029); use of low-dose TBI-based RIC (HR 1.16, 95 % CI 1.02–1.30; *p* = 0.019); and high CMV risk (seropositive recipient and seronegative donor) (HR 1.17, 95 % CI 1.03–1.33; *p* = 0.019). Karnofsky performance status at allo-SCT above 80 % was associated with improved LFS (HR 0.68, 95 % CI 0.57–0.81; *p* = 10^−5^) (Table 5).

### Overall survival

Overall survival at 2 years was 48.4 % (95 % CI 46.5–50.3). In univariate analysis, overall 2-year OS was significantly higher in HLA 10/10 M-URD group (50.6 %, 95 % CI 48.5–52.8) in comparison to mM-URD groups (41.3 %, 95 % CI 37.3–45.3 in HLA 9/10 and 43.5 %, 95 % CI 33.6–53.3 in HLA 8/10 mM-URD groups) (*p* = 0.0001) (Table 3). Improved OS with HLA 10/10 M-URD versus 9/10 and 8/10 mM-URD groups was observed in patients transplanted in CR1 (56.7 %, 95 % CI 53.8–59.6 versus 46.1 %, 95 % CI 40.1–52.2 and 50.2 %, 95 % CI 35.2–65.1, respectively, *p* = 0.005) but not in patients transplanted in ≥ CR2 or with active disease (Table 3, Figs. 2d and 3d). The use of in vivo T cell depletion had no impact on OS (*p* = 0.45) (Table 3). In multivariate analysis, the use of a 9/10 mM-URD was associated with reduced OS in comparison to HLA 10/10 M-URD (HR 1.27; 95 % CI, 1.13–1.44; *p* = 0.0001), while there was no difference between 9/10 and 8/10 mM-URD (*p* = 0.557) (Table 5). The other factors associated with shorter OS were age at SCT (HR 1.25; 95 % CI, 1.14–1.37; *p* < 10^−5^), disease status ≥ CR2 at SCT (HR 1.20; 95 % CI, 1.03–1.39; *p* = 0.016), active disease at SCT (HR 1.63; 95 % CI, 1.44–1.84; *p* < 10^−5^), secondary AML (HR 1.14; 95 % CI, 1.02–1.28; *p* = 0.021), use of low-dose TBI-based RIC (HR 1.16; 95 % CI, 1.02–1.32; *p* = 0.022), and high CMV risk (seropositive recipient and seronegative donor) (HR 1.25; 95 % CI, 1.09–1.43; *p* = 0.002). Karnofsky performance status at SCT above 80 % was associated with prolonged OS (HR 0.63; 95 % CI, 0.58–0.76; *p* < 10^−5^) (Table 5).

## Discussion

Unrelated donors represent the most frequent stem cell source for allo-SCT in Europe and are used in more than 50 % of RIC allo-SCT for AML patients [15]. In the absence of HLA-matched URD, a significant proportion of patients is transplanted with a 9/10 and to a less extends, with an 8/10 mM-URD. Although outcomes of RIC allo-SCT with HLA 10/10 or 8/8 MUD have been reported as comparable to transplants performed with a matched related donor [6, 18–20], the outcomes of M-URD versus mM-URD for older adults with AML have been poorly explored. This large, multicenter, registry study showed superior outcome using HLA-matched (10/10) donor compared to mM-URD (9/10 or 8/10) allo-SCT for AML in patients above the age of 50 years. Patients receiving mM-URD had significantly higher incidence of acute GVHD (both grades II–IV and II–IV) and NRM. A larger series of patients transplanted with RIC or MAC regimens for AML reported by the CIBMTR similarly showed increased risk of NRM with 7/8 mM-URD (*n* = 406) compared to 8/8 M-URD (*n* = 1193) or MRD (*n* = 624) due to increased incidence of acute GVHD in M-URD versus MRD [29].

We did not observe any impact of HLA matching on the overall incidence of chronic GVHD. However, in univariate analysis, the use of an 8/10 mM-URD was associated with increased risk of overall and extensive chronic GVHD particularly for patients transplanted in advanced phase disease. In our study, the other factors associated with a higher risk of both acute and chronic GVHD were active disease at transplantation and the absence of in vivo T cell depletion. In addition, patient age above 60 years and Karnofsky performance status below 80 % had a negative impact on NRM. Thus, the choice of a mM-URD for elderly patients transplanted with RIC-allo SCT requires the use of ATG and should take into consideration the higher risk of GVHD and NRM, in particular for patients above 60 years, not in CR and/or with an decreased performance status at allo-SCT.

In contrast to the reduced risk of relapse observed in the CIBMTR study with the use of 7/8 mM-URD in comparison to 8/8 MRD and M-URD [29], the use of a 9/10 mM-URD in our study was associated with a higher risk of relapse in comparison to 10/10 M-URD in multivariate analysis. There was, however, a trend for reduced relapse incidence with 8/10 in comparison to 9/10 mM-URD (HR 0.67, 95 % CI 0.43–1.02; *p* = 0.064). These differences might be explained by higher proportions of patients transplanted with active disease in the mM-MUD groups and by more intensive and prolonged immunosuppression administered to patients receiving mM-URD allo-SCT in order to control acute GVHD. This might have affected the development of the graft-versus-leukemia (GVL) effect, otherwise expected at higher level with mismatched donor T cells. Consistent with this hypothesis is the absence of increased chronic GVHD in the 9/10 mM-URD versus the 10/10 M-URD groups, while higher incidence of extensive chronic GVHD in the 8/10 mM-URD was associated with a trend towards reduced relapse incidence, in particular observed in patients transplanted in CR1. Another factor associated with increased risk of relapse, leading to reduced LFS and OS, was the use of a low-dose TBI-based RIC (truly non-ablative) regimen, suggesting that increasing the intensity of the conditioning might help for the control of the disease until the GVL response takes place [30].

As expected, disease status at SCT was another important factor impacting RI, LFS, and OS. In our study in older adults with AML transplanted in CR1, the 2-year OS was comparable to prior reports using RIC and MRD [11] and to younger AML patients transplanted with MAC regimens and MRD or M-URD allo-SCT [31]. Although LFS and OS were reduced with mM-URD in patients transplanted in CR, 2-year OS for patients transplanted in CR1 or in >CR1 were between 46 and 50 % with a 9/10 or 8/10 mM-URD transplantation, comparable to results of RIC for AML with MRD and M-URD [3, 4, 6], suggesting that mM-URD remains a valid option for AML above 50 years old, in particular for those transplanted in >CR1.

The feasibility of haplo-identical SCT performed with T replete stem cell grafts and in vivo T cell depletion based either on ATG [32] or post-transplant cyclophosphamide [33] has been demonstrated in the past 10 years. Comparisons of haplo-identical SCT using the Chinese approach combining T replete G-CSF mobilized bone marrow stem cell graft, a myeloablative conditioning regimen with ATG, to matched related and unrelated allo-SCT for acute leukemias have shown equivalent OS with reduced risk of relapse in high-risk leukemias in the haplo-identical groups [34, 35]. Using this approach, the Beijing’s group recently reported similar outcomes in fit patients transplanted above 50 years of age in comparison to younger patients [36]. In the setting of post-transplant cyclophosphamide, Blaise et al. reported in patients older than 60 years inferior outcomes of RIC allo-SCT performed with HLA 10/10 and 9/10 URD compared to those transplanted with MRD or haplo-identical donors because of higher NRM related to higher incidence of acute and chronic GVHD [37]. Comparisons of haplo-identical SCT at the era of post-transplant cyclophosphamide to URD have shown similar LFS and OS but reduced NRM and chronic GVHD with T replete haplo-SCT [38–41]. From 2012 onward, there has been increasing numbers of transplants performed from related haplo-identical donor, which is likely mainly due to increased use of haplo-identical donors with the post-transplant cyclophosphamide strategy. In AML patients undergoing allo-SCT without an HLA-matched (related or unrelated) donor, the decision to use one alternative graft source over another is complex. Published data support any one of the three alternative donor allo-SCT options (i.e., mM-URD, CBT, related haplo-identical) currently available for patients without a matched donor. Our current study support this notion as results of mM-URD in AML patients with age above 50 years transplanted in CR1 were inferior to matched URD and therefore other alternative like Haplo-SCT and CBT may be considered.

## Conclusions

We recognize that this study has limitations mainly due to the fact that it is a retrospective and registry-based study. Despite these limitations, these results suggest that HLA 10/10 M-URD is the preferable option for AML patients older than 50 years undergoing allogeneic transplantation following RIC preparative regimen. The use of a 9/10 or 8/10 mM-URD could be an alternative therapeutic option for patients not having a matched donor. Prospective randomized studies comparing mM-URD to other alternative donors, in particular haplo-identical SCT, are warranted.

## Abbreviations

aGVHD, acute graft-versus-host disease; Allo-SCT, allogeneic hematopoietic stem cell transplantation; AML, acute myeloid leukemia; ATG, anti-thymocyte globulin; BM, bone marrow; cGVHD, chronic graft-versus-host-disease; CMV, cytomegalovirus; CR, complete remission; LFS, leukemia-free survival; MAC, myeloablative conditioning; mM-URD, mismatched unrelated donor (); MRD, matched related donor; M-URD, matched unrelated donor; NRM, non relapse mortality (); OS, overall survival; PB, peripheral blood; RI, relapse incidence, RIC, reduced intensity conditioning; SOS, sinusoidal obstructive syndrome (); TBI, total body irradiation

## Additional file

Additional file 1: Table S1. List of institutions reporting patients’ data for the study. (DOCX 29 kb)
