# Supplementary material for: The impact of HLA-matching on reduced intensity conditioning regimen unrelated donor allogeneic stem cell transplantation for acute myeloid leukemia in patients above 50 years—a report from the EBMT acute leukemia working party
Source: J Hematol Oncol. 2016 Aug 3;9:65. doi: 10.1186/s13045-016-0295-9 (PMC4971653; doi:10.1186/s13045-016-0295-9)
Supplement: Additional file 1: Table S1. — List of institutions reporting patients’ data for the study. (DOCX 29 kb) [file 13045_2016_295_MOESM1_ESM.docx]

**Supplementary Table 1: List of institutions reporting patients’ data for the study**

| **EBMT centre** | N |
| --- | --- |
| 389 Leipzig [Univ, Haemat/Oncol] | 158 |
| 295 Hannover [Medical Univ] | 104 |
| 311 Wiesbaden [Kl Diagnostik] | 103 |
| 808 Dresden [Universitaets Kl] | 100 |
| 810 Freiburg [University] | 84 |
| 807 Berlin [Charite Univ] | 78 |
| 387 Birmingham [Queen Elizabeth] | 72 |
| 614 Hamburg [Univ H] | 71 |
| 534 Cologne [Univ, Medicine] | 70 |
| 718 Pilsen [Charles Univ H] | 59 |
| 153 Hamburg [AK St Georg] | 58 |
| 206 Copenhagen [Rigshospitalet] | 56 |
| 246 Rotterdam [Erasmus ` den Hoed] | 56 |
| 256 Kiel [UKSH] | 52 |
| 223 Tübingen [UnivTubingen] | 51 |
| 717 Nottingham [City H] | 50 |
| 239 Utrecht [University] | 46 |
| 267 Pessac [H Haut-Leveque] | 43 |
| 386 Bristol [Royal H Sick Children] | 42 |
| 204 Ulm [Medizin Kl / Polikl] | 41 |
| 524 Heidelberg [Medizinishce Kl] | 39 |
| 218 London [Royal Marsden] | 38 |
| 253 Nantes [Hotel Dieu] | 37 |
| 230 Marseille [Paoli Calmettes] | 36 |
| 207 Paris [St Louis] | 35 |
| 726 Liege [University] | 35 |
| 763 London [Kings College H] | 35 |
| 787 Regensburg [University] | 35 |
| 565 Maastricht [Univ H] | 34 |
| 277 Lille [H Claude Huriez] | 33 |
| 624 Toulouse [H Purpan] | 33 |
| 209 Leuven [Univ H] | 32 |
| 212 Stockholm [Univ H] | 31 |
| 704 Southampton [General H] | 31 |
| 270 Grenoble [H A Michallon] | 30 |
| 566 Cambridge [Addenbrookes H] | 29 |
| 588 Amsterdam [VU Univ Med Ctr] | 29 |
| 671 Lyon [H E Herriot] | 28 |
| 672 Strasbourg [H Hautepierre] | 28 |
| 676 Vandoeuvre_Les_Nancy [H d`Enfants] | 28 |
| 926 Montpellier [University] | 28 |
| 785 Homburg [Univ Saarland] | 26 |
| 303 Cardiff [Univ Wales] | 25 |
| 145 Stuttgart [Robert Bosch Kh] | 24 |
| 259 Essen [Univ H] | 24 |
| 261 Geneva [261] | 24 |
| 283 Lund [Univ H] | 24 |
| 813 Milano [S Raffaele] | 24 |
| 227 Vienna [Medizinische Univ] | 23 |
| 273 Clermont-Ferrand [Jean Perrin] | 22 |
| 513 Munich [Kl Grosshadern] | 22 |
| 634 Aarhus [Univ, Hematol] | 22 |
| 266 Uppsala [Univ H] | 21 |
| 308 Graz [Medical Univ] | 21 |
| 725 St._Petersburg [Pavlov Med Univ] | 21 |
| 786 Mainz [Johannes-Gutenberg] | 21 |
| 533 Jena [Friedrich-Schiller] | 20 |
| 552 Gottingen [Universitätskl] | 18 |
| 244 Glasgow [Royal Infirmary] | 17 |
| 252 Creteil [H Mondor Hematol] | 17 |
| 289 Goeteborg [Sahlgrenska Univ H] | 17 |
| 601 Manchester [Royal Infirmary] | 17 |
| 202 Basel [202] | 16 |
| 234 Brussels [St. Luc] | 16 |
| 740 Linköping [Univ H] | 16 |
| 809 Erlangen [University] | 16 |
| 208 Zürich [208] | 15 |
| 240 Bologna [S Orsola-Malpighi] | 15 |
| 680 Münster [University] | 15 |
| 731 Umeå [Univ H] | 15 |
| 977 Limoges [CHRU] | 15 |
| 597 Brno [Univ H] | 14 |
| 659 Brest [C.H.R.U Brest] | 14 |
| 233 Besancon [H Jean Minjoz] | 13 |
| 251 Caen [Hopital, Hematol] | 13 |
| 264 Poitiers [H La Miletrie] | 13 |
| 290 Karlsruhe [Klinikum] | 13 |
| 345 Haifa [Rambam MCH] | 13 |
| 656 Prague [Ist Hematology] | 13 |
| 712 Würzburg [Medizinische Kl II] | 13 |
| 729 Hradec_Králové [Charles Univ H, Hematol] | 13 |
| 941 Rouen [Becquerel] | 13 |
| 996 Antwerp_Edegem [UZA] | 13 |
| 224 London [UCL] | 12 |
| 284 Birmingham [Heartlands H] | 12 |
| 286 Pavia [S Matteo] | 12 |
| 339 Antwerp [AZ Stuivenberg] | 12 |
| 506 Brugge [AZ Sint-Jan] | 12 |
| 152 Augsburg [Zentra Kl] | 11 |
| 255 Oxford [Radcliffe H] | 11 |
| 294 Milano [Osp Niguarda] | 11 |
| 523 Nice [H de l`ARCHET I] | 11 |
| 644 Vilnius [Santariskiy Kl] | 11 |
| 768 London [S Bartholomew`s] | 11 |
| 778 Sheffield [Royal Hallamshire] | 11 |
| 215 Brussels [Jules Bordet] | 10 |
| 250 Saint_Etienne [St Etienne] | 10 |
| 515 Helsinki [Univ Central H] | 10 |
| 780 Manchester [Christie] | 10 |
| 203 Leiden [Univ H] | 9 |
| 546 Groningen [Univ H] | 9 |
| 677 Katowice [Silesian Med Acad] | 9 |
| 784 Essen [Evangelisches Kh] | 9 |
| 797 Vicenza [Osp S Bartolo] | 9 |
| 214 Barcelona [H Clinic] | 8 |
| 258 Jerusalem [Univ Hadassah] | 8 |
| 558 Munich [Rechts des Isar] | 8 |
| 590 Berlin [Benjamin Franklin] | 8 |
| 658 Bergamo [Ospedale, ematol] | 8 |
| 775 Paris [St Antoine] | 8 |
| 141 Brescia [Civili, Adulti] | 7 |
| 225 Turku [University] | 7 |
| 231 Torino [S. Giovanni (CTO)] | 7 |
| 257 Dublin [St James] | 7 |
| 260 Barcelona [SCreu i S Pau] | 7 |
| 271 Innsbruck [Univ H] | 7 |
| 299 Bolzano [Osp S Maurizio] | 7 |
| 530 Greifswald [Ernst-Moritz-Arndt] | 7 |
| 727 Salamanca [H Clinico] | 7 |
| 825 Alessandria [SS Antonio e Biagio] | 7 |
| 216 London [Royal Free] | 6 |
| 237 Nijmegen [St Radboud] | 6 |
| 248 Pescara [Osp Civile] | 6 |
| 409 Petach-Tikva [Beilinson H] | 6 |
| 538 Wroclaw [Ctr Cell Transpl] | 6 |
| 574 Olomouc [Univ H] | 6 |
| 598 San_Sebastian [H Aranzazu] | 6 |
| 705 Udine [Univ H] | 6 |
| 713 Leicester [Royal Infirmary] | 6 |
| 205 London [Hammersmith] | 5 |
| 235 Oslo [Rikshospitalet] | 5 |
| 302 Zagreb [Univ H Rebro] | 5 |
| 359 Magdeburg [vGuericke U] | 5 |
| 650 Angers [CHRU] | 5 |
| 744 Gent [Univ H] | 5 |
| 756 Rome [Tor Vergata] | 5 |
| 759 Barcelona [H Univ Bellvitge] | 5 |
| 300 Lisboa [Inst Oncologia] | 4 |
| 304 Firenze [Careggi-Meyer] | 4 |
| 338 Halle [Univ Martin-Luther] | 4 |
| 556 Budapest [National Med Ctr] | 4 |
| 592 Idar-Oberstein [Kl Knochenmarktr] | 4 |
| 594 Linz [Elisabethinen H] | 4 |
| 595 Melbourne [Alfred H] | 4 |
| 645 Marburg [Philipps Univ] | 4 |
| 769 Sevilla [Virgen del Rocio] | 4 |
| 970 Flensburg [St Franziskus] | 4 |
| 160 Paris [H Necker] | 3 |
| 211 Sao_Paulo [H Sirio-Libanes] | 3 |
| 305 Torino [Regina Margherita] | 3 |
| 613 Barcelona [H Trias i Pujol] | 3 |
| 617 Ankara [Ibni Sina H] | 3 |
| 625 Nürnberg [Klinikum] | 3 |
| 649 Bari [Univ Studi] | 3 |
| 661 Rennes [H Sud/Pontchaillou] | 3 |
| 954 Warsaw [Central] | 3 |
| 119 Ascoli_Piceno [Osp Mazzoni] | 2 |
| 161 Tel_Aviv [Sourasky] | 2 |
| 265 Milano [Osp Maggiore] | 2 |
| 354 Milano [Trapianto Midollo Osseo] | 2 |
| 392 Palermo [Osp V Cervello] | 2 |
| 526 San_Giovanni_Rotondo [IRCCS] | 2 |
| 561 Thessaloniki [G Papanicolaou G H] | 2 |
| 584 Barcelona [V d`Hebron Adults] | 2 |
| 602 Bremen [Kl Bremen-Mitte] | 2 |
| 606 Cuneo [S Croce e Carle] | 2 |
| 623 Verona [Policlinico] | 2 |
| 630 Brussels [Univ H] | 2 |
| 640 Ljubljana [Univ Med Ctr] | 2 |
| 665 Clamart [H Percy] | 2 |
| 666 Villejuif [Gustave Roussy] | 2 |
| 693 Warsaw [Inst Haematology] | 2 |
| 699 Wroclaw [Medical Acad] | 2 |
| 710 Perth [Royal H] | 2 |
| 754 Tel-Hashomer [Univ Adults] | 2 |
| 811 Cagliari [R Binaghi] | 2 |
| 930 Moscow [NRC Haem.] | 2 |
| 955 Amiens [H Sud] | 2 |
| 112 Hameln [KH Hameln-Pyrmont] | 1 |
| 232 Rome [Emat, `La Sapienza`] | 1 |
| 236 Madrid [Princesa] | 1 |
| 242 Santander [Valdecilla] | 1 |
| 254 Leeds [St James] | 1 |
| 287 Rome [S Camillo - Forlanini] | 1 |
| 291 Porto [Inst Oncologia] | 1 |
| 321 Siena [Le Scotte] | 1 |
| 390 Düsseldorf [Heinrich Heine Univ] | 1 |
| 427 Bucharest [Fundeni Clin Inst] | 1 |
| 501 Liverpool [Royal Univ H] | 1 |
| 539 London [St George`s] | 1 |
| 587 Reggio_Calabria [Centro Trapianti] | 1 |
| 610 Bratislava [Univ H] | 1 |
| 616 Milano [INT] | 1 |
| 652 Tricase_(Lecce) [C Panico] | 1 |
| 730 Poznan [K Marcinkowski Univ] | 1 |
| 734 Madrid [La Paz] | 1 |
| 735 Murcia [H M Meseguer] | 1 |
| 746 Tartu [Univ H] | 1 |
| 749 Oldenburg [Klinikum] | 1 |
| 766 Napoli [Federico II] | 1 |
| 788 Ancona [Umberto I] | 1 |
| 795 Pisa [Az Osp Univ] | 1 |
| 798 Christchurch [Canterbury Health] | 1 |
| 799 Gdansk [Medical U] | 1 |
| 819 Madrid [H G Marañón] | 1 |
| 919 Antalya [Medical Park H] | 1 |
| Total | 3398 |
